# Circulating miR-378 and miR-451 in serum are potential biomarkers for renal cell carcinoma

**DOI:** 10.1186/1479-5876-10-55

**Published:** 2012-03-22

**Authors:** Martina Redova, Alexandr Poprach, Jana Nekvindova, Robert Iliev, Lenka Radova, Radek Lakomy, Marek Svoboda, Rostislav Vyzula, Ondrej Slaby

**Affiliations:** 1 Masaryk Memorial Cancer Institute, Department of Comprehensive Cancer Care, Zluty kopec 7, Brno, Czech Republic; 2 Central European Institute of Technology, Masaryk University, Kamenice 5, Brno, Czech Republic; 3 Institute of Clinical Biochemistry and Diagnostics, Faculty of Medicine and Faculty Hospital in Hradec Kralove, Charles University, Hradec Kralove, Czech Republic; 4 Laboratory of Experimental Medicine, Institute of Molecular and Translational Medicine, Faculty of Medicine and Dentistry, Palacky University and Palacky University affiliated Hospital Olomouc, Brno, Czech Republic

**Keywords:** Renal cell carcinoma, MicroRNA, Serum, Biomarker

## Abstract

**Background:**

There is no standard serum biomarker used for diagnosis or early detection of recurrence for renal cell carcinoma (RCC) patients. MicroRNAs (miRNAs) are abundant and highly stable in blood serum, and have been recently described as powerful circulating biomarkers in a wide range of solid cancers. Our aim was to identify miRNA signature that can distinguish the blood serum of RCC patients and matched healthy controls and validate identified miRNAs as potential biomarkers for RCC.

**Methods:**

In the screening phase of the study, blood serum of 15 RCC patients and 12 matched healthy controls were analyzed by use of the TaqMan Low-Density Arrays enabling parallel identification of expression levels of 667 miRNAs through qRT-PCR-based approach. In the validation phase, identified miRNAs were further evaluated on the independent group of 90 RCC patients and 35 matched healthy controls by use of individual qRT-PCR assays and statistically evaluated.

**Results:**

We identified 30 miRNAs differentially expressed between serum of RCC patients and healthy controls: 19 miRNAs were up-regulated and 11 miRNAs were down-regulated in RCC patients. MiR-378, miR-451 and miR-150 were further evaluated in the independent group of patients, and two of them were successfully validated: levels of miR-378 were increased (p = 0.0003, AUC = 0.71), miR-451 levels were decreased (p < 0.0001, AUC = 0.77) in serum of RCC patients. Combination of miR-378 and miR-451 enable identification of RCC serum with the sensitivity of 81%, specificity 83% and AUC = 0.86.

**Conclusions:**

Circulating miRNAs in serum are promising biomarkers in RCC.

## Background

Renal cell carcinoma (RCC) is the most common neoplasm of adult kidney accounting for about 3% of adult malignancies and having the highest mortality rate at over 40% [1,2]. Renal tumors are commonly asymptomatic in early stages and although surgical resection remains the best therapy for RCC, after the curative nephrectomy, 20-40% patients will develop recurrence. Unfortunately, there is no standard serum biomarker used for diagnosis or early detection of recurrence for RCC patients. Although several serum proteins that might be useful to detect the presence of advanced or recurrent RCC have been reported [3], none indicated analytical sensitivity efficient enough for translation into standard of care management for RCC patients.

MicroRNAs (miRNAs) are a novel class of naturally occurring, short non-coding, single stranded RNAs, that regulate gene expression at the post-transcriptional level by binding to the untranslated region (3'UTR) of target mRNAs and causing translational inhibition and/or mRNA degradation [4]. Specific expression profiles of miRNAs have been shown in a variety of cancers, including RCC [4-6]. MiRNAs are highly stable and abundant in plasma, serum and other body fluids. Moreover, miRNA signatures in blood are similar in men and women, as well as individuals of different age [7]. These circulating miRNAs had shown great potential to serve as a novel biomarker for non-invasive diagnosis and prognosis of plenty kinds of diseases, such as cancer, and even physiological conditions such as prenatal screening. Following the release from cells, circulating miRNAs originate from (i) microvesicles (released by exocytosis), (ii) exosomes (formed via invagination of the early endosome and released upon fusion of late endosome with plasma membrane), and (iii) apoptotic vesicles and/or senescent bodies [7-9].

Expression profiles of circulating miRNAs were extensively studied in (i) colorectal cancer (CRC) where authors found miR-29a and miR-92 plasma levels to differentiate between CRC patients and controls with sensitivity of 89% and specificity of 70% and also to be associated with clinical stage [10], (ii) in ovarian cancer where levels of miR-21, miR-92 and miR-93 were higher in 3 patients with normal CA-125 levels, a clinical biomarker of ovarian cancer [11], (iii) in breast cancer where it has been described that changes in the level of total RNA, miR-10b, miR-34a, and miR-155 correlated with the presence of overt metastases [12], (iv) in prostate cancer where serum miR-141 levels were found to distinguish metastatic prostate patients from age-matched controls [13]. Such studies have clearly proved the potential of circulating miRNAs in primary diagnostics as well as in follow-up for the early detection of disease progression. In case of RCC the only study was performed and circulating miR-1233 identified, but the analytical characteristics of this biomarker in the validation study were not convincing [14]. In our study we focused on the circulating miRNAs expression profiles in the serum samples of RCC patients aiming identification of new biomarkers for RCC.

## Materials and methods

### Study population

We collected serum samples from the group of patients diagnosed for RCC and undergoing radical nephrectomy at Masaryk Memorial Cancer Institute (MMCI; Brno, Czech Republic) between 2003 and 2008, and matched cancer-free blood donor volunteers recruited from the same institute with no previous history of any cancer. All subjects were of the same ethnicity (Caucasian). Clinical and pathological characteristics including age, gender, stage, and grade were recorded and they are summarized in Table 1. RCC serum samples were collected after signing an informed consent prior to surgery and stored at MMCI Bank of Biological Material. The study has been approved by the local Ethical Committee at Masaryk Memorial Cancer Institute (MMCI; Brno, Czech Republic).

**Table 1:** Patient characteristics

	Exploratory phase		Validation phase	
	RCC	HC	RCC	HC
N	15	12	90	35
**Sex**				
male	10	10	56	26
female	5	2	34	9
**Age**				
median	62	61	66	63
range	53-73	54-69	36-85	51-70
**Histology**				
ccRCC	15		73	
pRCC	0		8	
chRCC	0		9	
**Pathological stage**				
pT1	6		50	
pT2	0		14	
pT3	9		23	
pT4	0		3	
vascular invasion	5		16	
lymph nodes metastatis	0		6	
distant metastasis	3		18	
**Fuhrman grade**				
G1	3		20	
G2	7		39	
G3	4		23	
G4	1		8	

*Abbreviatons: *RCC, renal cell carcinoma; ccRCC, clear cell RCC; pRCC, papillary RCC; chRCC, chromophobe RCC

### RNA isolation

Total RNA enriched for small RNAs was isolated using Qiagen miRNeasy Mini Kit (Qiagen, GmbH, Germany) from 200 μL serum according to modified manufacturers' protocol. For each sample, to the 800 μl of QIAzol solution was added 1.25 μl 0.8 μg/μl MS2 RNA carrier (Roche, cat. No. 10165948001). The extracted RNA was eluted in 50 μL of preheated Elution Solution, and concentration and purity of RNA were determined spectrofotometrically by measuring its optical density (A260/280 > 2.0; A260/230 > 1.8) using NanoDrop ND-1000 Spectrophotometer (Thermo Scientific, Wilmington, DE, USA). The samples were either stored at -80°C or further processed.

### TaqMan low density arrays - screening phase

In the screening phase, we performed TaqMan Low Density Arrays (TLDA) analysis to identify differentially expressed miRNAs from the two pooled serum samples (15 ccRCC patients vs. 12 healthy controls). In brief, 35 ng of total RNA was reverse-transcribed into cDNA by the TaqMan MicroRNA Reverse Transcription Kit and Megaplex RT set pool A and B version 2.0 (Applied Biosystems, CA, USA). To obtain sufficient amount of cDNA for TLDA analysis, pre-amplification step using TaqMan PreAmp MasterMix was added. The pre-amplified product was loaded into TaqMan Array Human MicroRNA A + B Cards Set v2.0 (Applied Biosystems, CA, USA) enabling simultaneous quantitation of 667 human miRNAs. TaqMan MicroRNA Assays and analysis were performed on the ABI 7900HT Instrument (Applied Biosystems, CA, USA). All reactions were performed according to the standard manufacturers' protocols. Quantitative miRNAs expression data were acquired and normalized by use of ABI 7900HT SDS software (Applied Biosystems, CA, USA).

### qRT-PCR - Validation phase

In validation phase of the study, candidate miRNAs identified by TLDA technology were further characterized. Complementary DNA (cDNA) was synthetised from total RNA using miRNA-specific primers according to the TaqMan MicroRNA Assay protocol (Applied Biosystems). For RT reactions 10 ng of RNA sample, 50 nM of stem-loop RT primer, 1 × RT buffer, 0.25 mM each of dNTPs, 3.33 U μl^-1 ^MultiScribe reverse transcriptase and 0.25 U μl^-1 ^RNase inhibitor (all from TaqMan MicroRNA Reverse Transcription kit, Applied Biosystems) was used. Reaction mixtures (15 μl) were incubated for 30 min at 16°C, 30 min at 42°C, 5 min at 85°C and then held at 4°C. qRT-PCR was performed using the Applied Biosystems 7500 instrument. The 20-μl PCR reaction mixture included 1.33 μl of RT product, 1 × TaqMan (AmpErase UNG) Universal PCR Master Mix and 1 μl of primer and probe mix of the TaqMan MicroRNA Assay kit (Applied Biosystems). Reactions were incubated in a 96-well optical plate at 50°C for 2 min, 95°C for 10 min, followed by 40 cycles at 95°C for 15 s and 60°C for 10 min. All reactions were run in duplicate and average threshold cycle and SD values were calculated.

### Statistical methods

In both phases, analysis of the qRT-PCR data was performed using the SDS 2.0.1 software (Applied Biosystems) (settings: automatic baseline, threshold 0.2). According to manufacturer's recommendations, miR-16 has been chosen as reference for normalization of miRNAs expression levels. The relative expression levels of target miRNAs were determined by the equation 2^-ΔCT^, in which ΔC_T _were calculated as follows: ΔC_T _= C_T miR-of-interest _- C_T miR-16_. Relative miRNA levels were then calculated with the RQ Manager 1.2. Normalized expression data from screening phase of the study were statistically evaluated in environment of statistical language R by use of Bioconductor package and LIMMA approach combined with hierarchical clustering (HCL) [15]. Normalized expression data in validation phase were statistically analyzed with MedCalc software version 11.2.1. P-values of less than 0.05 were considered statistically significant. Statistical differences between expression levels in RCC patients' and healthy controls' samples were evaluated by non-parametric Mann-Whitney U test. Sensitivity, specificity and area under curve (AUC) for serum microRNA levels were determined using Receiver Operator Characteristic (ROC) analysis.

## Results

### Screening phase

**Figure 1:**
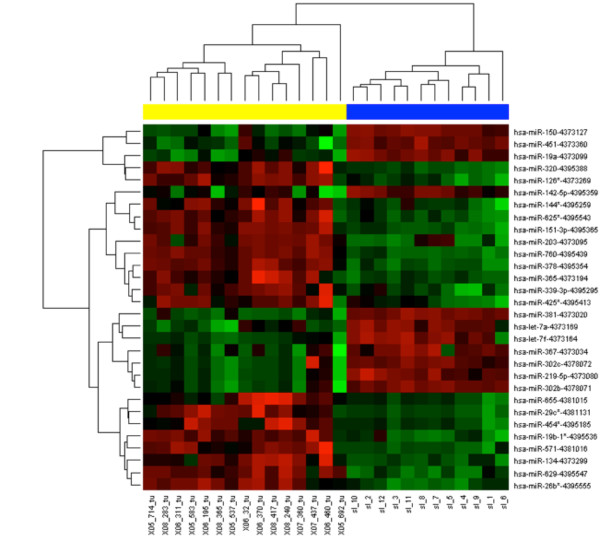
**Hierarchical clustergram iscriminating blood serum of RCC patients and healthy controls according to diferentially expressed miRNAs (yellow color indicate serum samples of RCC patients, blue healthy controls, p < 0.0001)**.

In this biomarker discovery phase, a strategy for effective identification of RCC-associated miRNAs in serum was performed using qRT-PCR-based miRNAs expression profiling arrays. We determined the miRNA expression profile of 667 miRNAs in serum from ccRCC patients (n = 15) using TaqMan Low Density Arrays technology, and compared to the expression profile of healthy individuals (n = 12); the miRNA expression levels were normalized to miR-16, the mean expression levels were calculated and data analyzed by use of the microarray biostatistical approaches. We observed 30 miRNAs differentially expressed between serum of ccRCC patients and healthy controls: 19 miRNAs were up-regulated in ccRCC patients and 11 miRNAs were down-regulated (Figure 1, Table 2). Among them, up-regulated miR-378 (FC = 37.6, p < 0.000001), and down-regulated miR-150 (FC = 0.02, p < 0.000001) and miR-451 (FC = 0.2, p < 0.000001) were proposed as putative biomarkers to the validation phase of the study, as significance of the difference (fold change, p-value), previous observations and biological plausibility (based on the PubMed hits when particular miRNA is combined with keyword "cancer"), and miRNAs with favorable expression levels (Ct > 30) were taken into account.

**Table 2:** MiRNAs diferentially expressed between serum of ccRCC patients and healthy controls

miRNA	Fold change	P-value	Probability, that gene is differentially expressed
hsa-miR-760	169.628	< 0.000001	100.000%
hsa-miR-381	0.002	< 0.000001	100.000%
**hsa-miR-378**	**37.596**	< 0.000001	**100.000%**
hsa-miR-151-3p	35.734	< 0.000001	100.000%
hsa-miR-629	45.790	< 0.000001	100.000%
**hsa-miR-150**	**0.020**	< 0.000001	**100.000%**
hsa-miR-219-5p	0.032	< 0.000001	99.999%
hsa-miR-26b*	41.191	< 0.000001	99.999%
hsa-miR-302b	0.057	< 0.000001	99.997%
hsa-miR-19b-1*	36.432	< 0.000001	99.994%
hsa-miR-126*	9.317	< 0.000001	99.972%
hsa-let-7f	0.011	< 0.000001	99.968%
hsa-miR-571	30.947	< 0.000001	99.978%
hsa-miR-29c*	23.842	< 0.000001	99.976%
hsa-miR-625*	13.286	< 0.000001	99.938%
hsa-let-7a	0.014	< 0.000001	99.922%
hsa-miR-134	40.828	< 0.000001	99.846%
hsa-miR-320	4.533	< 0.000001	99.811%
**hsa-miR-451**	**0.225**	< 0.000001	**99.723%**
hsa-miR-19a	0.261	< 0.000001	99.651%
hsa-miR-365	16.768	< 0.000001	99.642%
hsa-miR-339-3p	10.606	< 0.000001	99.524%
hsa-miR-203	79.565	< 0.000001	98.908%
hsa-miR-302c	0.044	< 0.000001	98.729%
hsa-miR-454*	11.965	< 0.000001	97.193%
hsa-miR-144*	6.425	< 0.00001	92.206%
hsa-miR-655	10.716	< 0.00001	84.080%
hsa-miR-367	0.133	< 0.00001	81.739%
hsa-miR-425*	6.666	< 0.0001	79.234%
hsa-miR-142-5p	0.136	< 0.0001	74.933%

*Abbreviations: *miR, microRNA/miRNA; ccRCC, clear cell renal cell carcinoma

### Validation phase

**Figure 2:**
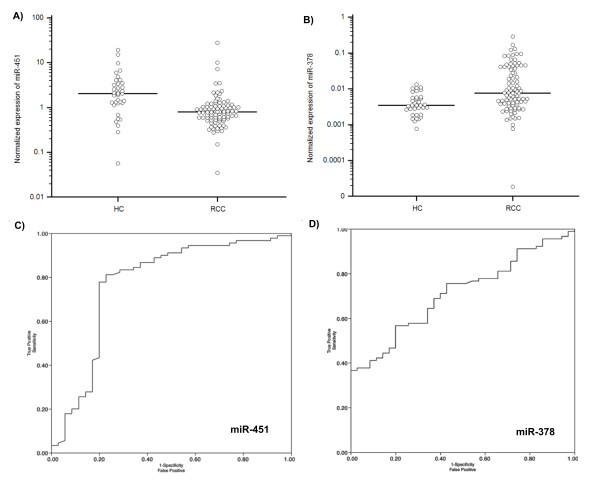
**Validation phase of miR-378, miR-451 and miR-150 in an independent group of serum samples (n = 125)**. Scatter plots of serum levels of (A) miR-378 and (B) miR-451 in healthy control (HC) samples (n = 35) and patients with RCC (n = 90). Expression levels of the miRNAs (log10 scale on the y-axis) are normalized to miR-16. The line represents the mean value. Statistically significant differences were determined using Mann-Whitney tests.

**Figure 3:**
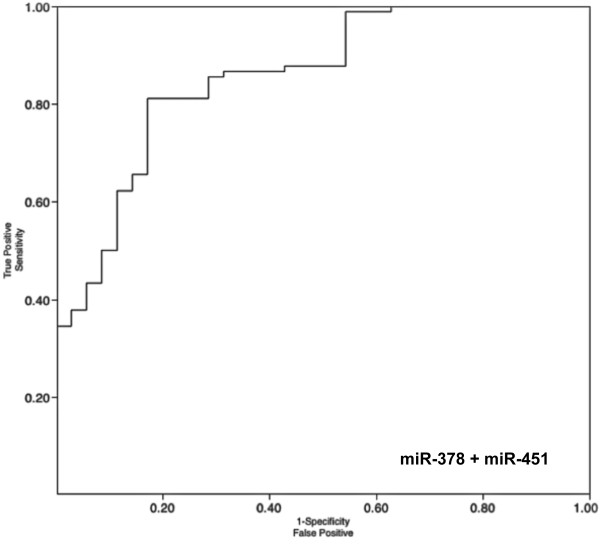
**ROC curve for combination of serum miR-378 and miR-451 yielded AUC of 0.86, the sensitivity of 81% and specificity of 83% in discriminating RCC**.

To validate the 3 promising biomarkers identified in the screening phase, miR-378, miR-451 and miR-150, qRT-PCR assays were developed to quantify miRNAs in serum of RCC patients and healthy controls. The qRT-PCR was performed in the independent cohort of 90 RCC patients' and 35 healthy controls' serum samples. As the difference in miR-150 expression level between RCC and healthy controls serum did not reach statistical significance (p = 0.222), miR-150 was excluded from further analysis. The expression of miR-378 serum level was significantly increased in patients with RCC compared to healthy controls (p = 0.0003), and the expression level of miR-451 was significantly decreased in patients with RCC compared to healthy controls (p < 0.0001), confirming the results of screening phase (Figure 2A-B, Table 3). Receiver operating characteristics (ROC) curve analysis revealed that the serum levels of both miR-378 and miR-451 might serve as useful biomarkers for differentiating serum of patients with RCC from controls with AUC of 0.71 (95% CI, 0.62 to 0.79), and 0.77 (95% CI, 0.69 to 0.84), respectively (Figure 2C-D). At the cut-off value of 0.02 for relative expression of miR-378 normalized to miR-16 levels, the sensitivity was 70% and the specificity was 60%. At the cut-off value of 2.0 for relative expression of miR-451 normalized to miR-16 levels, the sensitivity was 81% and the specificity was 77%. Multivariate logistic regression analysis showed that both miR-378 and miR-451 could serve as potential biomarkers for distinguishing between RCC and healthy controls, and even that their combination could improve the stratification power characterized with AUC of 0.86 and the sensitivity of 81% and specificity 83% (Figure 3).

**Table 3:** MiRNAs evaluated in the validation phase of study

miRNA	HC*	RCC*	p-value	Cancer association	Experimentaly validated target
miR-378	0.004 0.002-0.006	0.008 0.004-0.037	0.0003	colorectal carcinoma [16,17], oral squamous cell carcinoma [18], laryngeal carcinoma [19]	SUFU, TUSC2, TOB2, CYP2E1
miR-451	2.067 1.250-3.480	0.802 0.055-1.091	0.0001	renal cell carcinoma [1], colorectal carcinoma [20], gastric cancer [20]	MMP2, MMP9, BCL2
miR-150	0.011 0.009-0.016	0.008 0.005-0.020	0.2222	gastric cancer [21], chronic myeloid leukemia [22], colorectal carcinoma [23]	HTT, MYB, EGFR2

*Abbreviations: *HC, healthy controls; RCC, renal cell carcinoma

*expression levels are presented as medians and interquartile range

Receiver operating characteristics (ROC) curve analysis using (C) serum miR-451 yielded an AUC of 0.77, sensitivity of 81%, and specificity of 77% in discriminating RCC and (D) serum miR-378 yielded AUC of 0.71, sensitivity of 70%, and specificity of 60% in discriminating RCC

## Discussion

Many recent studies have described miRNAs expression profile in RCC and adjacent non-tumoral tissue confirming the different pattern. Although significant overlap between miRNAs identified by independent studies exist, regarding the number and type of up-/down-regulated miRNAs, data are in part conflicting [1,2,24,25]. Circulating miRNAs may possibly serve as a new class of powerful and non-invasive biomarkers for RCC patients. Regarding this hypothesis, we compared the miRNAs expression profiles of RCC patients' and healthy controls' serum samples. Based on the qRT-PCR arrays approach we were able to describe 30 differentially expressed miRNAs, 19 of these miRNAs were up-regulated and 11 were down-regulated. Such expression profile is a bit controversial, as the global miRNA down-regulation in RCC tissue samples has been repeatedly described [1-4]. The possible relation of miRNA levels between tissue and corresponding serum is still not clearly understood - some researchers postulate that circulating miRNAs may not always be directly associated with the changes occuring in tumor tissues but may also reflect indirect effects and could be secreted by non-tumoral cells. Also mechanisms were described how extracellular miRNAs can potentially interact with recipient cells via a number of different processes, including: direct fusion, internalization or receptor-mediated interactions. There are likely to be other mechanisms, especially for vesicle-free miRNAs, but all await further investigation to provide convincing evidence of their involvement in inter-cellular miRNA exchange [26,27]. However, this proposed potential of tumor cells to actively uptake miRNAs from circulation can partly explain the opposite trends of miRNA expression levels in tissue compared to blood serum. Several dysregulated miRNAs identified in our study have been described to have altered expression levels in plasma or serum of various cancers recently: up-regulated mir-425* [28,29], let-7a [30] or let-7f [31].

In the validation phase of this study, we tested 3 candidate miRNAs (miR-378, miR-451, miR-150) in the independent cohort of RCC patients. The up-regulation of miR-378 and down-regulation of miR-451 expression levels between serum of RCC patients and healthy controls reached statistical significance in validation study. Analytical characteristics of miR-378 (sensitivity of 70%, specificity of 60%) and miR-451 (sensitivity of 81%, specificity of 77%) proved that both miR-378 and miR-451 are potent in discriminating RCC from healthy control serum. Furthermore, combination of serum miR-378 and miR-451 levels, yielded sensitivity of 81% and specificity 83%, proved to be even more powerful discrimination tool. To our knowledge, the only study concerning circulating miRNAs in renal cell carcinoma identified circulating miR-1233 as a potential biomarker for RCC patients, but although they performed the validation phase on a large cohort of RCC patients from three different study centers, the diagnostic information was below their expectations (AUC of 0.588, sensitivity of 77%, specificity of 37.6%) [14].

Although our observations are promising, and miR-378/miR-451 analytical characteristics reached values for clinical utility, large-scale prospective studies aiming their evaluation in the renal benign neoplasms and early stages of the RCC are necessary to validate them as biomarkers for early diagnosis, and the analysis of blood serum collections from each patient to evaluate miRNA biomarker dynamics are needed to prove their potential for early detection of relapse in RCC patients.

## Conclusions

There is no standard serum biomarker used for diagnosis or early detection of recurrence for renal cell carcinoma (RCC) patients. In our study, we identified 30 miRNAs differentially expressed between serum of RCC patients and healthy controls. MiR-378, miR-451 and miR-150 were further evaluated in the independent group of patients, and two of them were successfully validated: levels of miR-378 were increased, miR-451 levels were decreased in serum of RCC patients. Combination of miR-378 and miR-451 enable identification of RCC serum with the sensitivity of 81%, specificity 83% and AUC = 0.86. We believe, that circulating miRNAs in serum are promising biomarkers in RCC.

## Competing interests

The authors declare that they have no competing interests.

## Authors' contributions

OS, MS and RV contributed to the conception and design of the study. AP, RL and MS were responsible for recruiting/supplying patients for the study. JN, OS, RI and LR were all involved with the acquisition and interpretation/analysis of study data. All the authors contributed to drafting and reviewing the manuscript, and all the authors read and approved the final manuscript.
